# Valorisation of Water Potabilization Sludges as Precursors for Alkali-Activated Binders: Characterization and Feasibility Study

**DOI:** 10.3390/ma16051998

**Published:** 2023-02-28

**Authors:** Marina Clausi, Daniela Pinto

**Affiliations:** Dipartimento di Scienze della Terra e Geoambientali, Università degli Studi di Bari Aldo Moro, 70121 Bari, Italy

**Keywords:** alkali activation, characterization, pre-treatments, valorisation, water potabilization sludge

## Abstract

Water potabilization sludges (WPS) are a heterogeneous waste generated from the coagulation–flocculation process of drinking water production, whose composition is highly dependent on the geological context of reservoirs, the composition and volume of treated water, and the types of coagulants used. For this reason, any feasible approach for reusing and valorising of such waste cannot be disregarded from the detailed investigation of its chemical and physical characteristics and they have to be evaluated at a local scale. In this study, WPS samples from two plants serving the Apulian territory (Southern Italy) were subjected for the first time to a detailed characterization with a view to evaluating their recovery and reuse at a local scale as a raw material for producing alkali activated binders. WPS samples were investigated by X-ray fluorescence (XRF), X-ray powder diffraction (XRPD) including phase quantification by the combined Rietveld and reference intensity ratio (RIR) methods, thermogravimetric and differential thermal analysis (TG-DTA), Fourier-transform infrared spectroscopy (FTIR) and scanning electron microscopy with energy dispersive X-ray spectroscopy (SEM-EDX). Samples showed aluminium–silicate compositions with up to 37 wt% of Al_2_O_3_ and up to 28 wt% of SiO_2_. Small amounts of CaO were also found (6.8 and 4 wt%, respectively). The mineralogical investigation indicates the presence of illite and kaolinite as clayey crystalline phases (up to 18 wt% and 4 wt%, respectively), in addition to quartz (up to 4 wt%) and calcite (up to 6wt%) and a large amorphous fraction (63 wt% and 76 wt%, respectively). WPS were subjected to heating from 400 °C to 900 °C and mechanical treatment by high energy vibro-milling in order to determine the best pre-treatment condition in view of their use as solid precursors to prepare alkali-activated binders. Alkali activation (8M NaOH solution; room temperature curing) was attempted on untreated WPS, on 700 °C heated and on 10-minute high-energy milled samples, which were considered the most suitable based on the preliminary characterization. Investigations of alkali-activated binders confirmed the geopolymerisation reaction occurrence. Variations in gel features and compositions depended on the amount of reactive SiO_2_, Al_2_O_3_ and CaO available in the precursors. WPS heated at 700 °C led to the most dense and homogeneous microstructures, due to a greater availability of reactive phases. The results of this preliminary study demonstrate the technical feasibility of preparing alternative binders from the investigated Apulian WPS, paving the way for a local reuse of these waste products, leading to economic and environmental benefits.

## 1. Introduction

Water potabilization sludges (WPS) are industrial by-products generated from the coagulation–flocculation process of drinking water production [1].

Owing to the constant need for drinking water, huge amounts of WPS are produced worldwide and are usually disposed of in landfills (European CER Cod 190902). To date, the challenge toward more sustainable waste management has promoted the reuse of WPS as ceramic materials, construction binders, zeolites and a coagulant in wastewater treatment [1,2,3,4,5,6]. With the same aim, several studies have focused on the use of WPS as precursors in the production of alkali-activated binders (AABs), leading to different results depending on the composition and eventual pre-treatments, as well as the mix design used [7,8,9,10,11,12,13]. 

AABs or geopolymers comprise a family of low carbon footprint materials obtained by the chemical combination of a structurally disordered aluminosilicate precursor with an alkaline activator able to catalyse the conversion of the blend into a compact matrix [14,15,16]. The precursor composition affects the gel type developments: a three-dimensional alkaline aluminosilicate hydrate gel M-A-S-H (M = alkaline cation) in low-calcium precursors, a two-dimensional calcium aluminosilicate hydrate gel C-A-S-H in Ca-rich precursors. A third type of binder, known as a hybrid alkaline cement, resulting from the alkaline activation of materials with around 20% CaO, SiO_2_ and Al_2_O_3_ contents, is formed by a mix of gels, (C, N)-A-S-H or N-(C)-A-S-H. [17,18]. 

In recent years, numerous studies have focused on the use of several kinds of industrial by-products and wastes (e.g., furnace slag, fly ash, ceramic wastes, biomasses, demolition wastes, stone waste, soil wastes) for AABs preparation, as alternatives to natural precursors (i.e., clays) [15,19,20,21,22]. 

Alkali-activated materials have been mainly studied as substitutes of traditional construction binders (e.g., Portland cement); however, different applications are nowadays fairly explored [14,15,16,17,18,19,20,21,22,23].

The high aluminium content of WPS, related to Al-based agents commonly added into the water to promote the particles aggregation, as well as their aluminosilicate nature [24], make this waste a potentially good solid precursor for AABs. However, the characteristics of WPS depend highly on the geological context of reservoirs, the water characteristics and the volume of treated water, along with the treatment processes, especially the types of coagulants used [8,25]. Today, the composition of WPS on a worldwide scale is fairly heterogeneous, mostly of aluminosilicate components [7,9,11,13,26], influencing the physical and chemical characteristics of the formulated materials derived from this kind of waste and making, in some cases, the comparison of literature data difficult. Thus, the detailed characterization of the sludge from local plants represents a fundamental prerequisite for any feasible approach for reusing and valorising such waste at a local scale as a raw material for AABs preparation.

In this paper, WPS from two different Italian plants serving the Apulian territory (Southern Italy) were for the first time fully characterized from a mineralogical, microstructural and chemical point of view by different techniques (namely, XRPD, XRF, TG-DTA, FTIR, SEM-EDS), and successively tested to assess their potential for the preparation of alkali activation binders and to find out the most suitable activation conditions for this purpose. 

Furthermore, two elements of novelty were introduced in this study with respect to previous ones dealing with the same topic [7,8,9,10,11,12,13]: (i) WPS were subject to different pre-treatments, i.e., thermal and mechanical, to evaluate the effects of heating and fine grinding, respectively, on the alkali-activated reaction products, as these two different pre-treatments may enhance the precursor characteristics to different degrees during alkali activation; (ii) In view of greater sustainability, alkali activation was promoted by using exclusively sodium hydroxide solutions, instead of commonly used alkaline silicate solution, which is quite expensive.

Overall, the research addresses transforming WPS into added value products, in order to stimulate their local valorisation, promoting a virtuous approach by which low-impact and tailor-designed materials are conceived based on the local availability of recycled resources. In this context the present study acquires high relevance as it explores for the first time the possibility to reuse WPS from Apulia (southern Italy region). The results of this study can in any case have a broader value on the investigated topic.

## 2. Materials and Methods

### 2.1. Characterization of Raw Materials

**Figure 1:**
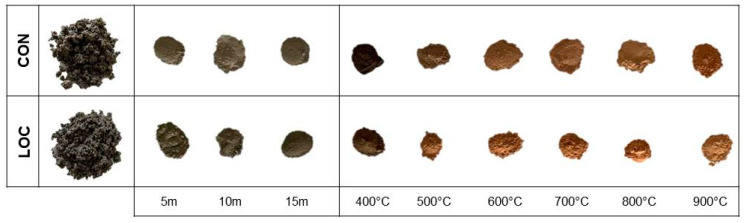
Features of dried CON and LOC sludge; appearances of sludges after the mechanical or thermal pre-treatments.

The WPS comes from two plants serving the Apulian territory (Southern Italy), sited in Conza della Campania and Locone (Minervino Murge). WPS (labelled CON and LOC, respectively) appeared as brownish granular aggregates of variable shapes and dimensions (within few cm) and were stabilized by drying at 105 °C for 24 h before being used (Figure 1). Mechanical or thermal pre-treatments according to the methodology described in [27] were experimented on to increase the reactivity of WPS to the alkaline-activation purpose: i.e., (i) high energy milling with a Retsch MM-400 vibratory mill (Retsch, Haan, Germany) and tungsten carbide grinding jars, frequency 30 Hz, t = 5, 10 and 15 min; (ii) heating for 1h at temperatures of 400, 500, 600, 700, 800 and 900 °C. 

WPS samples were characterized using the following methodologies:

- X-ray fluorescence (XRF) spectroscopy using a PANalytical AXIOS Advanced (PANalytical, Almelo, The Netherlands)—spectrometer, equipped with an X-ray tube X SST-mAX, Rh anode, with determination of volatile components by Loss on ignition (L.o.I.).
- Thermogravimetric and differential thermal analysis (TG-DTA-DTG) using a Seiko thermal analyzer SSC 5200 combined with a TG/DTA 320U module (Seiko, New Castle, DE, USA). Analyses were performed on about 20 mg of previously stabilized powder, in the temperature range 26–1100 °C (heating rate 10 °C/min).
- X-ray powder diffraction (XRPD) spectroscopy using a PANalytical Empyrean diffractometer (PIXcel3D detector; Cu-Kα radiation) (Malvern PANalytical, Almelo, The Netherlands); analytical conditions: 40 mA, 40 kV, 3–70° 2θ, virtual scan 0.026°, counting time 120 s. X-ray patterns were analysed using the software X’Pert High Score 3.0e (Malvern PANalytical, Almelo, the Netherland) which includes the ICSD database, whereas phase quantification was carried out by means of the Rietveld refinement software BGMN (J.Bergmann, Dresden, Germany) [28], using the internal corundum standard to quantify the amorphous phase according to the combined Rietveld-RIR method [29].
- Fourier-transform infrared spectroscopy (FT-IR) with a Perkin Elmer ATR spectrometer (PerkinElmer Italia Spa, Milano, Italy), recording 16 interferometer scans per sample from 400 to 4000 cm^−1^, nominal resolution of 2 cm^−1^ and data interval 0.25 cm^−1^.
- Stereomicroscopic acquisitions using a Nikon SMZ800 (Nikon Corp., Tokyo, Japan) stereomicroscope, magnification range 10× and 60×.
- Scanning electron microscopy LEO model EVO-50XVP (Zeiss, Cambridge, Cambridgeshire, UK), coupled with an X-max (80 mm^2^) Silicon drift Oxford detector EDX system (SEM-EDX) (Oxford Instruments, High Wycombe, Buckinghamshire, UK).

### 2.2. Samples Preparation

AABs were prepared by mixing WPS with 8M NaOH solution. Viscous pastes were obtained using an alkali solution / precursor weight ratio (S/P) from 0.6 to 0.8. The slurries were mixed for 5 min using a mechanical mixer, poured into moulds and vibrated to remove the entrained air. Samples were cured at 25 °C in sealed vessels for 3 days, then demoulded and characterized by XRPD, FT-IR and SEM-EDX at 28 days of curing using the above-described instruments and analytical conditions. 

## 3. Results and Discussion

### 3.1. Precursors Characterization

CON and LOC show aluminosilicate compositions (Table 1) with total amounts of SiO_2_ and Al_2_O_3_ ranging from 55 to 60 wt%, and SiO_2_/Al_2_O_3_ molar ratio of 1.73 and 1.04, respectively. The mass losses after 24 h at 105 °C were about 80 wt%, whereas from 105–950 °C (loss on ignition range), they were 31 wt%. The dominant chemical components (SiO_2_ and Al_2_O_3_) are quite different to those found in other investigations [7,8,9,10,12,13,26]: in particular, LOC exhibits the larger amount of Al_2_O_3_. 

**Figure 2:**
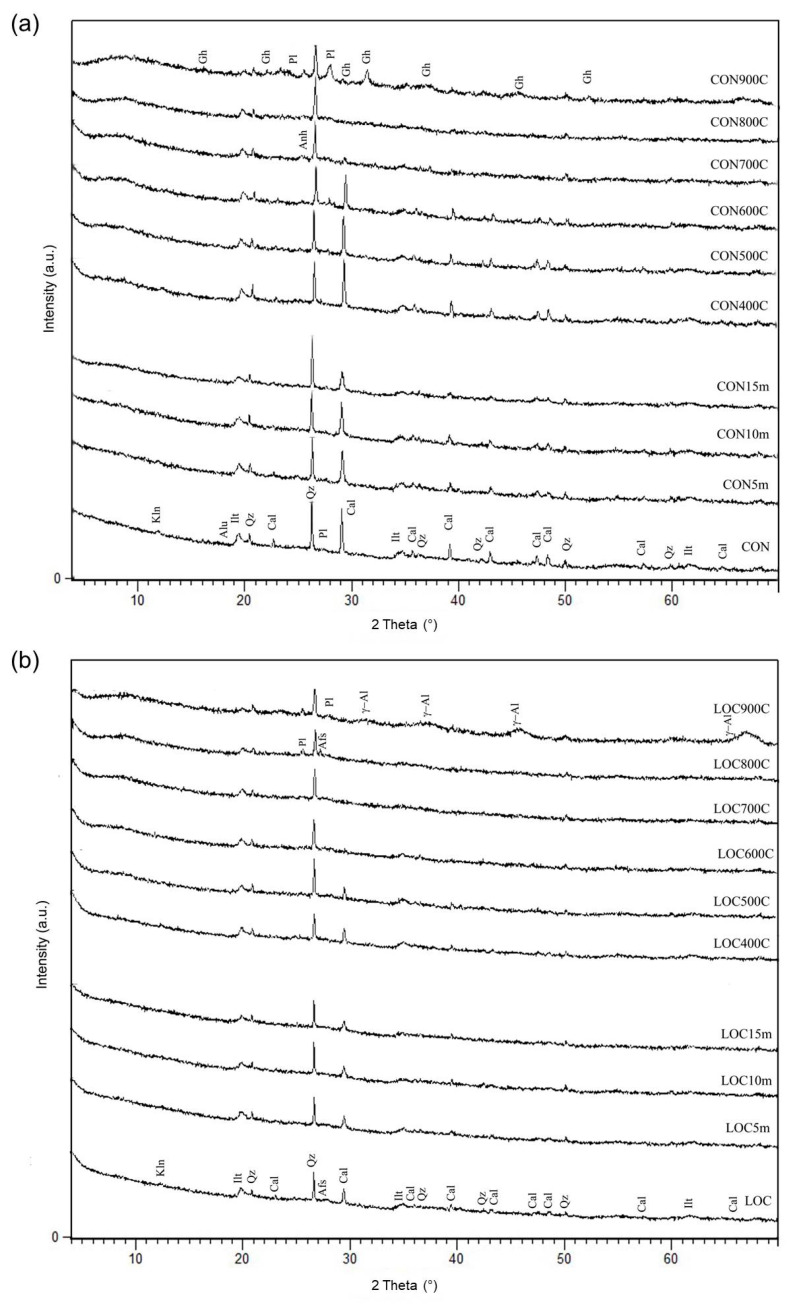
XRPD patterns of untreated and pre-treated WPS: (**a**) CON and (**b**) LOC. Afs = alkali feldspar; Alu = Alunogen; Anh = Anhydrite; Cal = Calcite; Gh = Gehlenite; Ilt = Illite; Kln = Kaolinite; Pl = Plagioclase; Qz = Quartz; γ-Al: γ-Alumina. The peaks that are not indexed are the same as those indicated in XRPD patterns of the starting material.

The investigated WPS are composed by crystalline phases of illite, kaolinite, quartz and calcite with subordinate plagioclase, K-feldspar and alunogen (bottom patterns of Figure 2). The presence of alunogen, observed in CON, can be ascribable to coagulants used in the water processing plant (i.e., alum (Al_2_(SO_4_)_3_)) [30]. 

Phase quantification by means of Rietveld refinement evidenced the significant amount of amorphous phase of 63.5% and 75.9% for CON and LOC, respectively (Table 2). Considering the total amount of Al, estimated from the Al-bearing crystalline phases, it can be concluded that a significant content of Al is related to the occurrence of amorphous or low crystalline phases [31].

**Figure 3:**
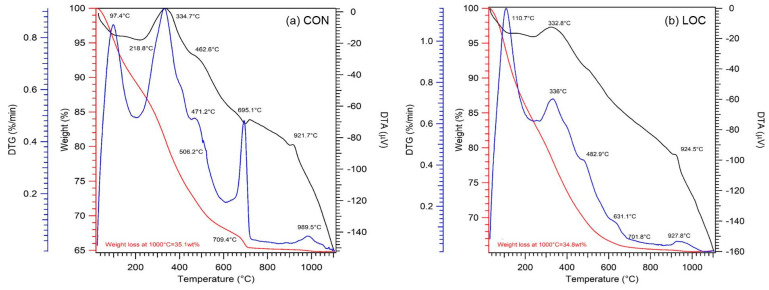
TG (red lines; left axis), DTA (black lines; right axis) and DTG (blue lines; external axis) curves of (**a**) CON and (**b**) LOC.

Figure 3 shows thermal analyses of CON and LOC. Total weight losses of about 35%, mainly within 710 °C, were recorded for both WPS, indicating a significant occurrence of volatile components (i.e., organic matter, hydrated phases and carbonates). Indeed, DTA curves show several peaks, similar in both WPS, reported as follows. Endothermic peaks in the range 97–230 °C were related to physically adsorbed water or weakly bound water. The weight losses observed in this range varied within 13–15%. Strongly exothermic peaks at about 330 °C were attributed to decomposition of the organic fraction [7,25] and result in weight losses of about 13%. In the range 400–600 °C, the aluminosilicates species decomposition was observed, notably the endothermic peaks at 460 °C indicate the dehydroxylation of kaolinite with the subsequent metakaolin formation [32]. The endothermic peaks at about 920 °C can be attributed to the phase transformation of clay minerals [30,32]. In CON an endothermic peak at 695 °C related to carbonates decomposition and resulting in a weight loss of 1.5% was also observed. The slight differences in shape and position of the endothermic peaks of each crystalline component, compared to those reported in the literature, may be related to a grain size reduction or lower structural order in the material, induced during the manufacturing process (from reservoir water sampling until the sludge removal) [33,34].

**Figure 4:**
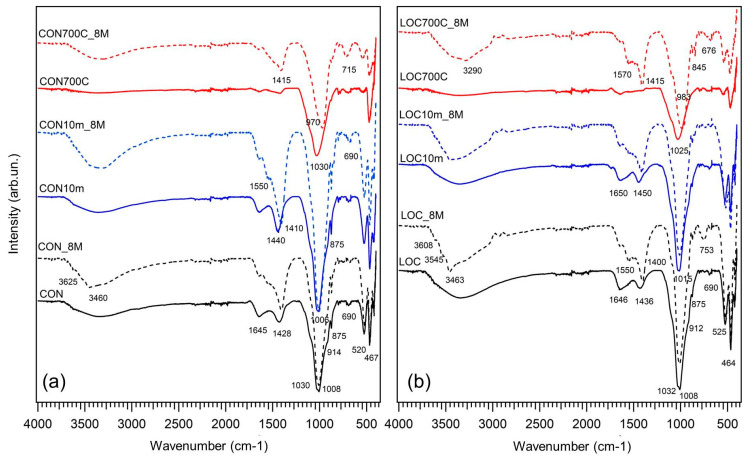
FTIR spectra of WPS used as precursors (solid-curve) and AABs (dotted-curve): (**a**) CON and (**b**) LOC.

FT-IR spectra (bottom of Figure 4) show the distinctive aluminosilicates band centred at 1008 cm^−1^ enveloping the different T-O-T (T = Al or Si) contributions (shoulders between 900 and 1100 cm^−1^) of silicate minerals [35] present in the samples. Other silicate vibrations are associated with the bands at about 520 and 465 cm^−1^ [36]. The broad band at 3350 cm^−1^, the humps at 1645 and about 520 cm^−1^ correspond, respectively, to the bending and stretching vibrations of the -OH groups in interlayer water molecules. ${\mathrm{CO}}_{3}^{2-}$ group vibrations of carbonates are revealed at about 1430 cm^−1^ and 875 cm^−1^. 

**Figure 5:**
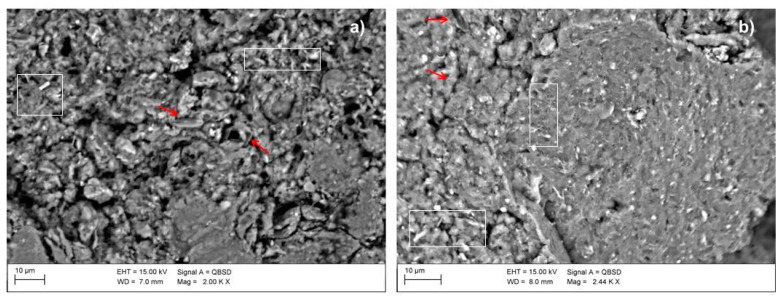
SEM micrographs of (**a**) CON and (**b**) LOC. Scale bar and magnification are shown in the image. White squares and red arrows evidence the presence of phyllosilicates and microfossils, respectively.

SEM images of precursors (Figure 5) show fairly homogeneous microstructures formed by different size clusters with a rough texture, which create hollow areas among the paired particles. In particular, in LOC, the coexistence of a more compact area (right part of Figure 5b) with a less bonded one was observed. Both samples evidence a slightly crystalline appearance [1,25] in which lamellar shapes of phyllosilicates (indicated by squares in the image) and some siliceous microfossils (indicated by arrows) are still recognizable in accordance with XRPD analyses. The EDX microanalysis (graphs not reported) returned fairly constant compositions in the different areas analysed, consisting mainly of Si, Al, Ca and in minor amounts, K, Mg and Fe. 

### 3.2. Thermal and Mechanical Pre-treatments on Precursors

XRPD patterns of thermally and mechanically pre-treated WPS are shown in Figure 2. Heating from 400 °C to 500 °C did not produce significant variations in the mineral compositions with the exception of the kaolinite dehydroxylation. At 600 °C, calcite starts to decompose and the reaction is completed at 700 °C. In CON, the presence of anhydrite (CaSO_4_) was also detected. At 800 °C, illite and quartz are still detectable, and in addition, plagioclase formations were also noted in LOC. New phases are observed at 900 °C, although different in two cases: plagioclase (anorthite) and gehlenite in CON, plagioclase and γ-Al_2_O_3_ in LOC. 

Based on TG-DTA analysis and mineralogical investigation of thermal-treated samples, the heating temperature of 700 °C was selected as the most suitable for AABs preparation, as it produces the complete dehydroxylation of kaolin and calcite present, preserving the amorphous nature of the sludges. This result is in fair agreement with literature data. Ferone et al. [7] suggested a calcination temperature of 650 °C for 1h in order to promote the thermal activation of clay minerals and the removal of the organic phases present. Messina et al. [8] and Santos et al. [10] fixed the temperature diversifying the calcination duration at 750 °C, whereas Waijarean et al. [26] considered the calcination at 800 °C the optimum pre-treatment for geopolymers manufacturing. The optimal burning temperatures were determined experimentally in each case, since they depend closely on the mineralogical composition. 

High energy milling at different times produced moderate effects on the crystalline phases of WPS. All components are still detectable; however, mild illite and kaolinite reflection widening, as the effect of delamination, was observed. 

The mechanical pre-treatment method is well-known to improve the reactivity of clay minerals [27,37,38,39]. The method allows transferring of as much energy as possible to the ground material to enhance its chemical reactivity [37]. Furthermore, the dissolution degree of precursors in an alkaline medium is facilitated by the large surface amorphism and particle size reduction achievable by high energy milling [40,41]. Considering that the crystalline variations observed by increasing the grinding time were not very significant, with a view to energy-saving an intermediate milling time (10 min) was chosen for the experimentation follow up in both CON and LOC. 

The quantification of the amorphous phase obtained by the combined Rietveld-RIR method (Table 2) indicates an increase of the amorphous after each selected treatment, with higher values in heat-treated samples, thus indicating a greater availability of the reactive phase in these samples.

### 3.3. Characterization and Properties of Hardened AABs

Untreated WPS, as well as pre-treated WPS samples, were used to test their suitability for the preparation of AABs. Sample labels indicate the treatment undergone and the activator solution molarity (i.e., LOC_8M, LOC10m_8M, LOC700C_8M).

**Figure 6:**
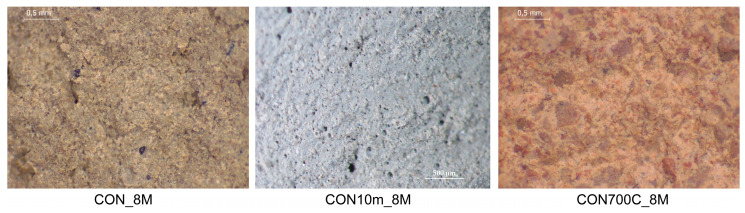
Stereomicroscope images from inner sections of CON-based AABs magnified by 30×.

On a visual inspection performed after 28 days of curing, samples appeared hardened and compact. Stereomicroscope images of CON-based samples (Figure 6) showed bonded and variously porous matrices, with no fractures or cracks. Similar shapes were observed in Santos et al. [10] for WPS-Portland cement-based geopolymers. Furthermore, no patinas were observed on the inner surfaces by high magnification investigation.

The occurrence of the geopolymerization reaction was confirmed by changes in the FTIR strongest aluminosilicates vibration (Figure 4). In heat-treated samples (CON700C_8M and LOC700C_8M), this band was clearly shifted to lower wavenumbers compared to those of respective precursors (from about 1025–1030 cm^−1^ to 970–980 cm^−1^), whereas in untreated and mechanically-treated samples, it took on a narrower shape, indicating a different structural order [42]. In addition, shifts observed in heat-treated samples may have been intensified by structural changes due to calcium, made available from calcite decarbonatation, which contributed to calcium-enriched N-A-S-H gel formation [9,43]. The main band related to carbonates (between 1415 cm^−1^ and 1440 cm^−1^) appeared more intense than the precursor ones, as a result of binders interaction with atmospheric CO_2_ during the curing process. Broad bands (within 3000 cm^−1^ and 3600 cm^−1^) corresponding to –OH groups were also revealed. In particular, in LOC-based samples and in CON_8M, three peaks can be distinguished at 3620, 3545 and 3460 cm^−1^. 

**Figure 7:**
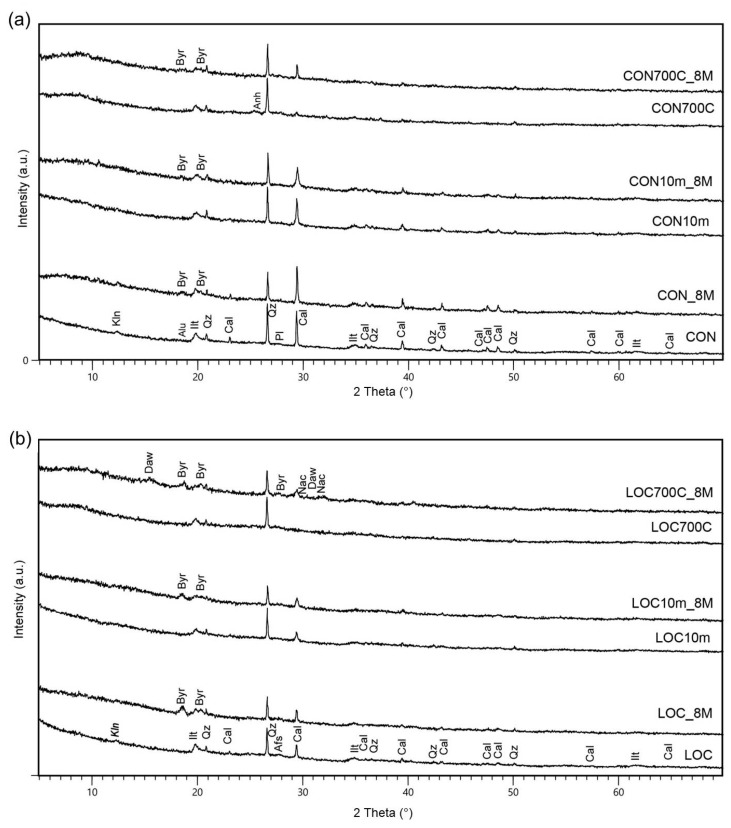
XRPD patterns of AABs (**a**) CON and (**b**) LOC. WPS used as precursors are reported as references. Afs = alkali feldspar; Alu = Alunogen; Anh = Anhydrite; Byr = Bayerite; Cal = Calcite; Daw = Dawsonite; Ilt = Illite; Kln = Kaolinite; Nac = Nahcolite; Pl = Plagioclase; Qz = Quartz.

CON_8M and LOC_8M diffractograms (Figure 7) showed the same peaks detected in raw materials as well as the presence of bayerite (Al(OH)_3_), probably formed as a consequence of an excessive availability of aluminium capable of reacting with OH^−^ in solution [31]. Similar observations can be drawn for CON10m_8M and LOC10m_8M. However, a slight decreasing of illite peaks (at 19.7° and 34.7° 2θ) was also noted due to crystalline delamination induced by grinding, which seems to have facilitated the dissolution process in NaOH. In heat-treated pastes, further peaks were distinguished: calcite (CaCO_3_) deriving from Ca interaction with atmospheric CO_2_, nahcolite (NaHCO_3_) and dawsonite (NaAl(CO_3_)(OH)_2_), the latter detected in LOC700_8M. The presence of sodium carbonates was attributed to the carbonation of the remnant alkalis supplied by the activator and present in the matrix [44]. As is well known [45,46,47,48], the growth of salts within the structure of hardened binders can potentially cause the structure weakening or reduce the samples’ integrity. Therefore, the obtained findings here suggest that further studies on the aforementioned samples may require the use of lower NaOH content.

**Figure 8:**
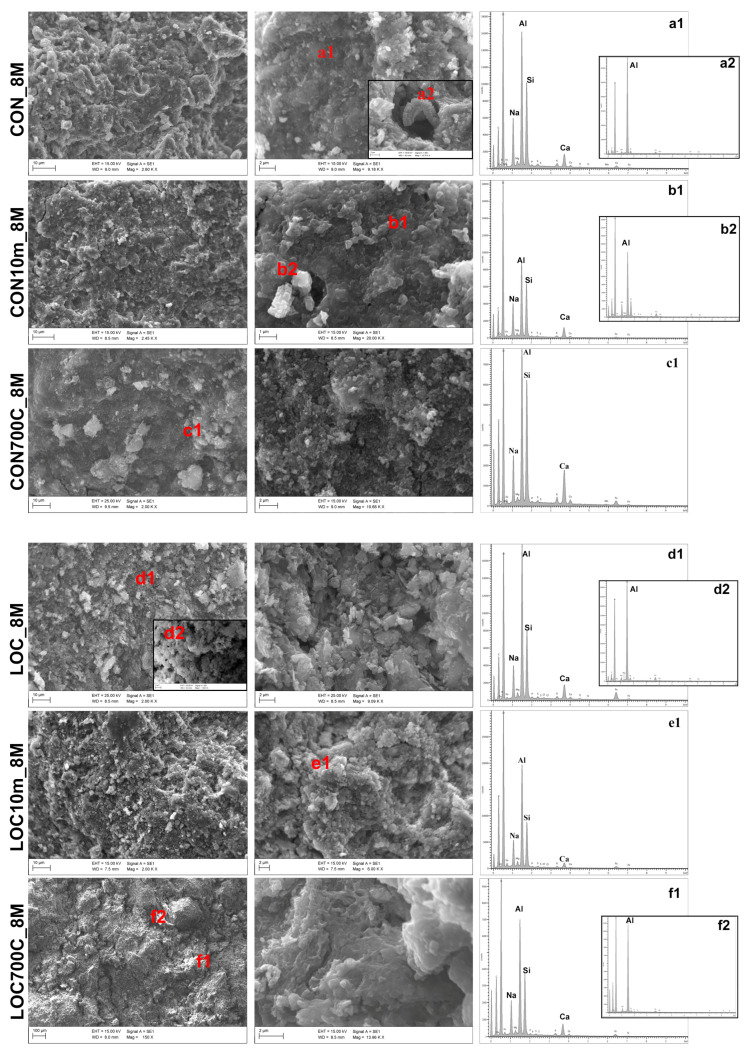
SEM micrographs and EDX analyses of AABs. Labels, scale bar and magnification are shown in the images.

SEM detections are reported in Figure 8. At low magnifications (left column in figure), all samples show developed and fairly homogeneous matrices, whereas some differences were observed at higher enlargements (right columns in figure). CON_8M and LOC_8M were characterized by rounded or slightly flat clusters (<2 µm), which appeared less bonded in the latter. Milling affects the gel features (CON10m_8M and LOC10m_8M ) by decreasing the size (<1 µm) and increasing the packaging of particles. AABs from un-treated and mechanically treated precursors are mostly constituted by Al-rich N-A-S-H gels (insets a1-b1-d1-e1). The aluminium tri-hydroxides crystalline phases, already revealed by XRPD, appeared as Al-formed lamellar aggregates (image points and EDX graphs a2-b2-d2-f2) [49]. Compositions of heat-treated samples were shifted towards ((N-C)-A-S-H) gels (insets c1-f1), due to the presence of reactive CaO (larger in CON700C_8M), that gave rise to the most compact and homogeneous microstructures. In these samples, moderate tapered terminations, distinctive of materials enriched in calcium, were distinguished. 

As a whole, the investigation furnishes interesting data. The SiO_2_/Al_2_O_3_ precursor molar ratios (Table 1) proved sufficient, albeit lower than the range defined by Duxson et al. [50], to allow alkali activation. The explanation may lie in the large amount of the Al-rich amorphous component. Likewise, results confirmed the NaOH 8M solution validity for geopolymerization purposes. Furthermore, mix designs adopted for CON and LOC avoid incurring long hardening times [26] or reduced workability [11]. Among the two investigated WPS, the higher SiO_2_/Al_2_O_3_ molar ratio and a greater availability of reactive calcium enabled obtaining a greater structural compactness of CON-based specimens than LOC-based ones. However, further studies should be oriented at modifying the SiO_2_/Al_2_O_3_ through the addition of natural or waste-derived silicon sources, to reduce secondary crystalline formations. 

Samples characterization highlighted the efficacy of both pre-treatments. Mechanical activation proved to be a feasible method which mainly influenced the compactness of particles. Nevertheless, as evidenced by TG-DTA analyses, about 13 wt% of whole LOC and CON was attributed to organic matter, which could gradually affect the technological properties of pastes, in agreement with Geraldo et al [11]. Calcinated sludges became more reactive, thus enhancing the final product features; in addition, high temperatures might allow to remove contaminations. In light of these findings, further studies may investigate in greater detail the physical–mechanical properties of AABs.

## 4. Conclusions

This work sought to characterize two water potabilization sludges from plants in Apulia, Italy with a view to evaluating their feasibility for local reuse as precursors of alkali-activated binders. The chemical and mineralogical characterization of WPS by XRPD, TG-DTA, FTIR and SEM defined their aluminosilicate-rich compositions, with total amounts of SiO_2_ and Al_2_O_3_ ranging from 55 to 60 wt%, and the presence of illite, kaolinite, quartz and calcite as the main crystalline phases. Phase quantification by means of Rietveld refinement highlighted the significant amount of amorphous phase (up to 76%). Furthermore, the occurrence of Al mainly as amorphous or low crystalline phases was determined.

With the aim of promoting an efficient use of WPS, thermal or mechanical pre-treatments were performed on the raw sludge. XRPD analyses enabled indication of how heating at 700 °C and the 10 min high-energy milling resulted in the most suitable pre-treatments.

AABs were produced using untreated and activated WPS. The success of the alkali activation reaction was demonstrated by the changes involved in FTIR spectra and by the development of matrices mainly constituted by Al-rich N-A-S-H or ((N-C)-A-S-H) gels. XRPD evidenced also secondary crystalline phases (i.e. carbonates and aluminium tri-hydroxides). As a whole, both the pre-treatment methods enabled enhancing the binder features; however, heating proved the most suitable since it allows obtaining of a larger availability of reactive phases and more compact microstructures.

This study represents an important starting point with a view to a local valorisation of these wastes. The findings can guide future investigations towards optimizing synthesis parameters in view of the applicability of the investigated WPS as raw materials for AABs preparation. Based on the microstructural and mineralogical characteristics of the preliminary pastes obtained here from untreated and treated WPS samples, an addition of Si-sources may be proposed to better balance mix-designs and reduce the development of secondary crystalline phases. With the same aim, lowering the activator molarity and testing solid and non-expensive activators, as well as better assessing the curing conditions, may provide positive outcomes. Furthermore, forthcoming studies will allow evaluating the mechanical and physical properties of pastes to define their eventual application fields.

As further conclusion, it is important to highlight the relevance of sourcing raw materials at a local scale. It may be an opportunity to valorise the territories and lowering the production costs. Moreover, the reuse of waste-derived resources, currently dismissed in landfills, such as WPS, may lead to economic and environmental benefits and contributing to the end-of-waste idea.

## Figures and Tables

**Table 1:** Bulk chemical (XRF) composition (wt%) of CON and LOC.

	CON	LOC
SiO_2_	28.00	22.87
Al_2_O_3_	27.42	37.30
CaO	6.87	4.03
Fe_2_O_3_	3.09	2.44
K_2_O	0.87	0.71
Na_2_O	0.14	0.17
MgO	1.01	0.81
TiO_2_	0.32	0.23
P_2_O_5_	0.31	0.37
MnO	0.29	0.11
L.o.I. ^a^	31.68	30.96
SiO_2_ + Al_2_O_3_	55.42	60.17
SiO_2_/Al_2_O_3_ molar ratio	1.73	1.04

^a^ L.o.I.: weight loss after calcination at 950 °C.

**Table 2:** Mineralogical composition (wt%) of untreated and pre-treated WPS obtained by Rietveld refinement. Standard deviation in parentheses.

	CON	CON10m	CON700C	LOC	LOC10m	LOC700C
quartz (SiO_2_)	4.4 (3)	4.0 (3)	5.9 (5)	3.0 (3)	2.9 (3)	3.1 (2)
calcite (CaCO_3_)	6.2 (2)	5.4 (3)	1.2 (2)	2.5 (2)	2.0 (2)	0.1 (1)
kaolinite (Al_2_Si_2_O_5_)(OH)_4_	4.1 (3)	3.9 (3)		3.4 (3)	2.4 (3)	
illite KAl_2_Si_4_O_10_(OH)_2_	18.3 (1.1)	14.1 (1.2)	15.7 (1.3)	14.6 (1)	11.3 (1)	14.0 (1.1)
K-feldspar (KAlSi_3_O_8_)				0.6 (1)	1.0 (2)	0.7 (2)
dolomite (CaMg(CO_3_)_2_)	tr	tr	tr			
plagioclase (Na,Ca)((Si,Al)AlSi_2_)O_8_	2.0 (2)	2.7 (3)	4.1 (4)			
alunogen Al_2_(SO_4_)_3_·17H_2_O	1.4 (3)	2.2 (4)				
anhydrite (CaSO_4_)			1.6 (2)			
amorph	63.5 (1.5)	67.6 (1.5)	71.6 (1.6)	75.9 (1.2)	80.4 (1.1)	82.0 (1.1)

## Data Availability

Further inquiries can be directed to the corresponding author.

## References

[1] Ahmad T., Ahmad K., Alam M. (2016). Sustainable management of water treatment sludge through 3‘R’ concept. J. Clean. Prod..

[2] Gomes S.D.C., Zhou J.L., Li W., Long G. (2019). Progress in manufacture and properties of construction materials incorporating water treatment sludge: A review. Resour. Conserv. Recycl..

[3] Babatunde A.O., Zhao Y.Q. (2007). Constructive Approaches Toward Water Treatment Works Sludge Management: An International Review of Beneficial Reuses. Crit. Rev. Environ. Sci. Technol..

[4] Cremades L., Cusidó J., Arteaga F. (2018). Recycling of sludge from drinking water treatment as ceramic material for the manufacture of tiles. J. Clean. Prod..

[5] Rozhkovskaya A., Rajapakse J., Millar G.J. (2020). Synthesis of high-quality zeolite LTA from alum sludge generated in drinking water treatment plants. J. Environ. Chem. Eng..

[6] Nair A.T., Ahammed M.M. (2015). The reuse of water treatment sludge as a coagulant for post-treatment of UASB reactor treating urban wastewater. J. Clean. Prod..

[7] Ferone C., Capasso I., Bonati A., Roviello G., Montagnaro F., Santoro L., Turco R., Cioffi R. (2019). Sustainable management of water potabilization sludge by means of geopolymers production. J. Clean. Prod..

[8] Messina F., Ferone C., Molino A., Roviello G., Colangelo F., Molino B., Cioffi R. (2017). Synergistic recycling of calcined clayey sediments and water potabilization sludge as geopolymer precursors: Upscaling from binders to precast paving cement-free bricks. Constr. Build. Mater..

[9] Mañosa J., Cerezo-Piñas M., Maldonado-Alameda A., Formosa J., Giro-Paloma J., Rosell J., Chimenos J. (2021). Water treatment sludge as precursor in non-dehydroxylated kaolin-based alkali-activated cements. Appl. Clay Sci..

[10] Santos G.Z., Filho J.A.M., Pinheiro M., Manzato L. (2019). Synthesis of water treatment sludge ash-based geopolymers in an Amazonian context. J. Environ. Manag..

[11] Geraldo R.H., Fernandes L.F., Camarini G. (2017). Water treatment sludge and rice husk ash to sustainable geopolymer production. J. Clean. Prod..

[12] Hwang C.-L., Chiang C.-H., Huynh T.-P., Vo D.-H., Jhang B.-J., Ngo S.-H. (2017). Properties of alkali-activated controlled low-strength material produced with waste water treatment sludge, fly ash, and slag. Constr. Build. Mater..

[13] Nimwinya E., Arjharn W., Horpibulsuk S., Phoo-Ngernkham T., Poowancum A. (2016). A sustainable calcined water treatment sludge and rice husk ash geopolymer. J. Clean. Prod..

[14] Palomo A., Maltseva O., Garcia-Lodeiro I., Fernández-Jiménez A. (2021). Portland Versus Alkaline Cement: Continuity or Clean Break: “A Key Decision for Global Sustainability”. Front. Chem..

[15] A Bernal S., Rodríguez E.D., Kirchheim A.P., Provis J.L. (2016). Management and valorisation of wastes through use in producing alkali-activated cement materials. J. Chem. Technol. Biotechnol..

[16] Provis J.L., Palomo A., Shi C. (2015). Advances in understanding alkali-activated materials. Cem. Concr. Res..

[17] Garcia-Lodeiro I., Palomo A., Fernández-Jiménez A., Macphee D. (2011). Compatibility studies between N-A-S-H and C-A-S-H gels. Study in the ternary diagram Na2O–CaO–Al2O3–SiO2–H2O. Cem. Concr. Res..

[18] Palomo A., Krivenko P.V., Garcia-Lodeiro I., Kavalerova E., Maltseva O., Fernández-Jimenez A.M. (2014). A review on alkaline activation: New analytical perspectives. Mater. Constr..

[19] Capasso I., Liguori B., Ferone C., Caputo D., Cioffi R. (2020). Strategies for the valorization of soil waste by geopolymer production: An overview. J. Clean. Prod..

[20] Rivera J., Castro F., Fernández-Jiménez A., Cristelo N. (2020). Alkali-Activated Cements from Urban, Mining and Agro-Industrial Waste: State-of-the-art and Opportunities. Waste Biomass- Valorization.

[21] D’Elia A., Clausi M., Fernández-Jiménez A., Palomo A., Eramo G., Laviano R., Pinto D. (2023). Alkali-Activated Binary Binders with Carbonate-Rich Illitic Clay. Polymers.

[22] Occhipinti R., Fernández-Jiménez A.M., Palomo A., Tarantino S.C., Riccardi M.P., Clausi M., Zema M. (2023). Effect of NaOH molarity on the formation of hybrid cements from sulfate-bearing clay and Pietra Serena sludge. Mater. Lett..

[23] Luukkonen T., Heponiemi A., Runtti H., Pesonen J., Yliniemi J., Lassi U. (2019). Application of alkali-activated materials for water and wastewater treatment: A review. Rev. Environ. Sci. Bio/Technol..

[24] Mañosa J., Formosa J., Giro-Paloma J., Maldonado-Alameda A., Quina M., Chimenos J. (2020). Valorisation of water treatment sludge for lightweight aggregate production. Constr. Build. Mater..

[25] de Godoy L.G.G., Rohden A.B., Garcez M.R., da Costa E.B., Da Dalt S., Andrade J.J.D.O. (2019). Valorization of water treatment sludge waste by application as supplementary cementitious material. Constr. Build. Mater..

[26] Waijarean N., Asavapisit S., Sombatsompop K. (2014). Strength and microstructure of water treatment residue-based geopolymers containing heavy metals. Constr. Build. Mater..

[27] D’Elia A., Pinto D., Eramo G., Giannossa L., Ventruti G., Laviano R. (2018). Effects of processing on the mineralogy and solubility of carbonate-rich clays for alkaline activation purpose: Mechanical, thermal activation in red/ox atmosphere and their combination. Appl. Clay Sci..

[28] Bergmann J., Friedel P., Kleeberg R. (1998). BGMN—A New Fundamental Parameters Based Rietveld Program for Laboratory X-ray Sources, Its Use in Quantitative Analysis and Structure Investigations. CPD Newsl..

[29] Gualtieri A.F. (2000). Accuracy of XRPD QPA Using the Combined Rietveld-RIR Method. J. Appl. Cryst..

[30] Ling Y.P., Tham R.-H., Lim S.-M., Fahim M., Ooi C.-H., Krishnan P., Matsumoto A., Yeoh F.-Y. (2017). Evaluation and reutilization of water sludge from fresh water processing plant as a green clay substituent. Appl. Clay Sci..

[31] Tantawy M. (2015). Characterization and pozzolanic properties of calcined alum sludge. Mater. Res. Bull..

[32] Gualtieri A., Bellotto M. (1998). Modelling the structure of the metastable phases in the reaction sequence kaolinite-mullite by X-ray scattering experiments. Phys. Chem. Miner..

[33] Tchakoute H., Rüscher C., Djobo J., Kenne B., Njopwouo D. (2015). Influence of gibbsite and quartz in kaolin on the properties of metakaolin-based geopolymer cements. Appl. Clay Sci..

[34] Pérez-Rodríguez J., Pascual J., Franco F., de Haro M.J., Duran A., del Valle V.R., Pérez-Maqueda L. (2005). The influence of ultrasound on the thermal behaviour of clay minerals. J. Eur. Ceram. Soc..

[35] Farmer V.C. (1974). The Infrared Spectra of Minerals; Mineralogical Society Monograph.

[36] Madejová J. (2003). FTIR techniques in clay mineral studies. Vib. Spectrosc..

[37] Tole I., Habermehl-Cwirzen K., Cwirzen A. (2019). Mechanochemical activation of natural clay minerals: An alternative to produce sustainable cementitious binders – review. Miner. Pet..

[38] MacKenzie K.J.D., Brew D.R.M., Fletcher R.A., Vagana R. (2007). Formation of aluminosilicate geopolymers from 1:1 layer-lattice minerals pre-treated by various methods: A comparative study. J. Mater. Sci..

[39] Mañosa J., Gómez-Carrera A., Svobodova-Sedlackova A., Maldonado-Alameda A., Fernández-Jiménez A., Chimenos J. (2022). Potential reactivity assessment of mechanically activated kaolin as alternative cement precursor. Appl. Clay Sci..

[40] Provis J.L. (2018). Alkali-activated materials. Cem. Concr. Res..

[41] Balczár I., Korim T., Kovács A., Makó É. (2016). Mechanochemical and thermal activation of kaolin for manufacturing geopolymer mortars—Comparative study. Ceram. Int..

[42] Lee W.K.W., van Deventer J.S.J. (2003). Use of Infrared Spectroscopy to Study Geopolymerization of Heterogeneous Amorphous Aluminosilicates. Langmuir.

[43] García-Lodeiro I., Jimenez A.M.F., Blanco-Varela M.T., Palomo A. (2007). FTIR study of the sol–gel synthesis of cementitious gels: C–S–H and N–A–S–H. J. Sol-Gel Sci. Technol..

[44] Bernal S.A., Provis J.L., Brice D.G., Kilcullen A., Duxson P., van Deventer J.S. (2012). Accelerated carbonation testing of alkali-activated binders significantly underestimates service life: The role of pore solution chemistry. Cem. Concr. Res..

[45] Longhi M.A., Zhang Z., Walkley B., Rodríguez E.D., Kirchheim A.P. (2021). Strategies for control and mitigation of efflorescence in metakaolin-based geopolymers. Cem. Concr. Res..

[46] Kani E.N., Allahverdi A., Provis J.L. (2012). Efflorescence control in geopolymer binders based on natural pozzolan. Cem. Concr. Compos..

[47] Zhang Z., Provis J.L., Reid A., Wang H. (2014). Fly ash-based geopolymers: The relationship between composition, pore structure and efflorescence. Cem. Concr. Res..

[48] Lee W., van Deventer J. (2002). The effects of inorganic salt contamination on the strength and durability of geopolymers. Colloids Surfaces A Physicochem. Eng. Asp..

[49] Zhang Y., Chang J., Zhao J., Fang Y. (2018). Nanostructural characterization of Al(OH) _3_ formed during the hydration of calcium sulfoaluminate cement. J. Am. Ceram. Soc..

[50] Duxson P., Provis J.L., Lukey G.C., Mallicoat S.W., Kriven W.M., van Deventer J.S.J. (2005). Understanding the relationship between geopolymer composition, microstructure and mechanical properties. Colloids Surf. A Physicochem. Eng. Asp..

